# Cryptococcal Meningitis Without Headache: A Case Report Highlighting an Atypical Presentation

**DOI:** 10.7759/cureus.92208

**Published:** 2025-09-13

**Authors:** Yvanne Joshua Rabe, Ailleen M Villegas, Valmarie S Estrada, Mary Shiela J Ariola-Ramos

**Affiliations:** 1 Section of Adult Neurology, Department of Internal Medicine, Cardinal Santos Medical Center, San Juan, PHL; 2 Department of Neurology, University of the East Ramon Magsaysay Memorial Medical Center, Quezon City, PHL; 3 Institute for Neurosciences, St. Luke's Medical Center, Taguig, PHL; 4 Department of Internal Medicine, Cardinal Santos Medical Center, San Juan, PHL

**Keywords:** atypical presentation, cryptococcal meningitis, disseminated cryptococcal infection, headache absence, steroid treatment

## Abstract

*Cryptococcus neoformans* meningoencephalitis is a prevalent manifestation of cryptococcosis and disseminated infection, frequently observed in immunosuppressed individuals and untreated acquired immunodeficiency syndrome (AIDS) patients. Prevalent causes of immunosuppression encompass glucocorticoid medication, organ transplants, malignancies, and various other disorders. Symptoms include headache, fever, vomiting, and altered mentation. We present a 61-year-old Filipino female diagnosed with autoimmune hemolytic anemia on high-dose glucocorticoid therapy who presented with dyspnea and febrile episodes. The initial workup suggested community-acquired pneumonia, but further testing with blood cultures revealed yeast cells, and the serum cryptococcal antigen titer was markedly elevated (1:4096). Even with the absence of headache or other neurological symptoms, a lumbar puncture was done due to a high index of suspicion, eventually revealing an elevated opening pressure of 51 cmH₂O and a positive CALAS (Cryptococcal Antigen Latex Agglutination System) in cerebrospinal fluid. The patient was then treated with liposomal amphotericin B and fluconazole as per treatment protocol, with serial lumbar punctures showing a gradual decline in antigen titers and intracranial pressure. She remained clinically stable and was transitioned to consolidation therapy with fluconazole. This case illustrates the importance of early CNS evaluation in immunocompromised patients with high cryptococcal antigen titers, even in the absence of classical symptoms. Cryptococcal meningitis should be identified in immunocompromised patients, especially when serum antigen titers are high. High-dose corticosteroids may mask symptoms of headache through multiple mechanisms of action, and the absence of symptoms should not exclude the diagnosis. Early diagnosis, comprehensive evaluations, and prompt antifungal treatment are crucial for improved patient outcomes.

## Introduction

*Cryptococcus neoformans* meningoencephalitis is the most common manifestation of cryptococcosis, particularly in disseminated infection. It is a serious opportunistic infection that occurs in immunosuppressed patients and patients with untreated AIDS [1]. The most common forms of immunosuppression, apart from HIV, include glucocorticoid therapy, solid organ transplantation, cancer (particularly hematologic malignancy), and other conditions such as sarcoidosis and hepatic failure. The most frequent underlying diseases, factors, and complications of cryptococcal meningitis patients are corticosteroid use, pulmonary infection, hepatobiliary diseases, and diabetes mellitus [2].

The term "meningoencephalitis" is more appropriate than "meningitis" since histopathologic examination has demonstrated that the brain parenchyma is almost always involved. In patients with cryptococcal meningitis, the most common initial symptom is headache, followed by fever, vomiting, and altered mentation [2]. This paper aims to illustrate that even patients with no symptoms but with laboratory-proven disseminated cryptococcal infection should have a high suspicion for central nervous system (CNS) involvement. Previously published case reports for atypical presentations of cryptococcal meningitis include headaches with associated dental pain, new-onset recurrent headaches in a 40-year-old female, and a 52-year-old HIV-seronegative patient with a five-year history of chronic headache [3-5]. There have been no recent reports of atypical presentations of cryptococcal meningitis that involve the absence of a headache.

## Case presentation

We present a case of a 61-year-old Filipino female diagnosed with autoimmune hemolytic anemia, receiving immunosuppression-glucocorticoid therapy, who was admitted to our institution due to difficulty breathing and febrile episodes. The workup revealed air bronchograms on a chest X-ray, and on a chest CT scan, a rounded, non-calcified parenchymal opacity was identified. An eventual CT-guided biopsy of the lung mass was performed. The patient was initially managed as a case of community-acquired pneumonia (moderate risk), given piperacillin-tazobactam and clindamycin, but blood cultures eventually revealed yeast cells. Biopsy revealed fungal spores using both Gram stain and Grocott’s methenamine silver stain. A serum cryptococcal antigen test was performed, which showed an elevated titer of 1:4096 dilution.

The patient denied any symptoms of headache, blurring of vision, vomiting, or altered mentation. Despite the absence of neurologic symptoms, the very high serum titer warranted a strong suspicion of central nervous system involvement. The neurologic physical examination was mostly unremarkable. The patient had an intact mental status examination, with no craniopathies or sensorimotor deficits, and with no neck rigidity. However, the fundoscopic examination, using only direct ophthalmoscopy, revealed an intact red-orange reflex and clear media, but it also showed cotton wool spots and blurred disk margins, which suggested increased intracranial pressure. Image of the fundus, however, was not taken at the time of examination due to the unavailability of a fundus camera.

**Figure 1:**
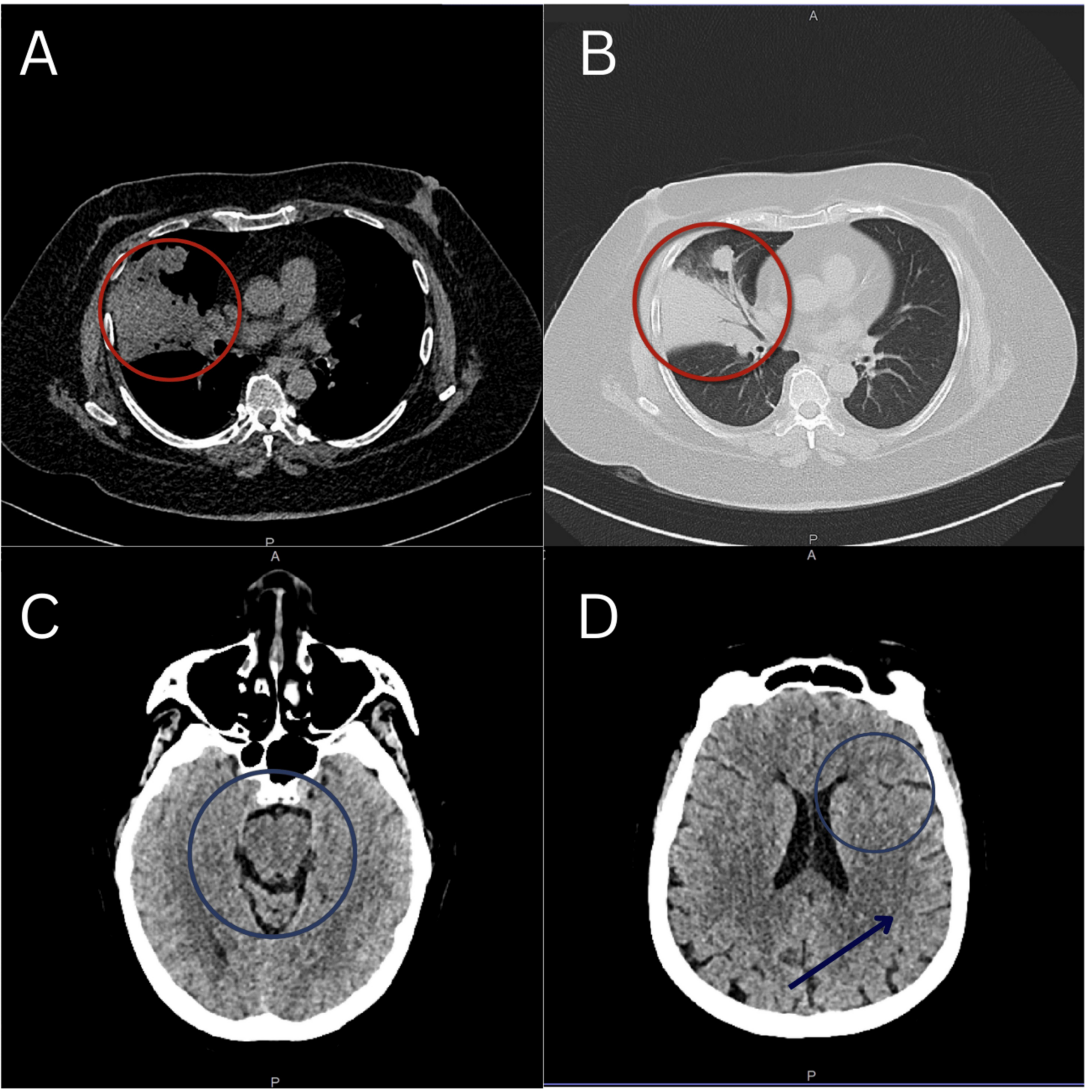
The patient's high-resolution plain chest (A and B) and cranial (C and D) CT scans. The red circles indicate the non-calcified parenchymal opacity seen in the high-resolution plain chest CT scan. The blue circle in image C shows the intact cisterns, and in image D, the intact sulci with no effacement. The blue arrow represents intact gray and white matter, indicating the absence of increased intracranial pressure.

The patient underwent cranial CT, then lumbar puncture, albeit traumatic, with an opening pressure of 51 cmH2O, and medical decompression was initiated using osmotic diuretic mannitol 20%, computed at 0.5 g/kg given every six hours. Cranial imaging was unremarkable (Figure 1). The initial routine CSF workup is shown in Table 1.

**Table 1:** Initial routine cerebrospinal fluid workup. CALAS: Cryptococcal Antigen Latex Agglutination System.

Test	Normal values	Result
Fluid description		Approximately 1 mL of slightly hazy, pale red fluid with no clot or pellicle
Red blood cell count	0	70 x 10^6^/L
White blood cell count	0	0 x 10^6^/L
Total cell count	<5	70 x 10^6^/L
Protein	150-450	462 mg/L
Sugar ratio to serum	~66% of blood	22%
Gram stain		Polymorphonuclear cells: rare. Yeast cell: few
India Ink		Positive for *Cryptococcus neoformans*
CALAS quantitative		Positive for cryptococcal antigen up to 1:1024
Anaerobic culture and sensitivity		No growth after 48 hours of anaerobic conditions
Aerobic culture and sensitivity		Isolate 1: *Cryptococcal neoformans* (very light growth). Sensitive to amphotericin B and flucytosine
Meningitis/encephalitis panel		*Cryptococcus neoformans* detected

**Figure 2:**
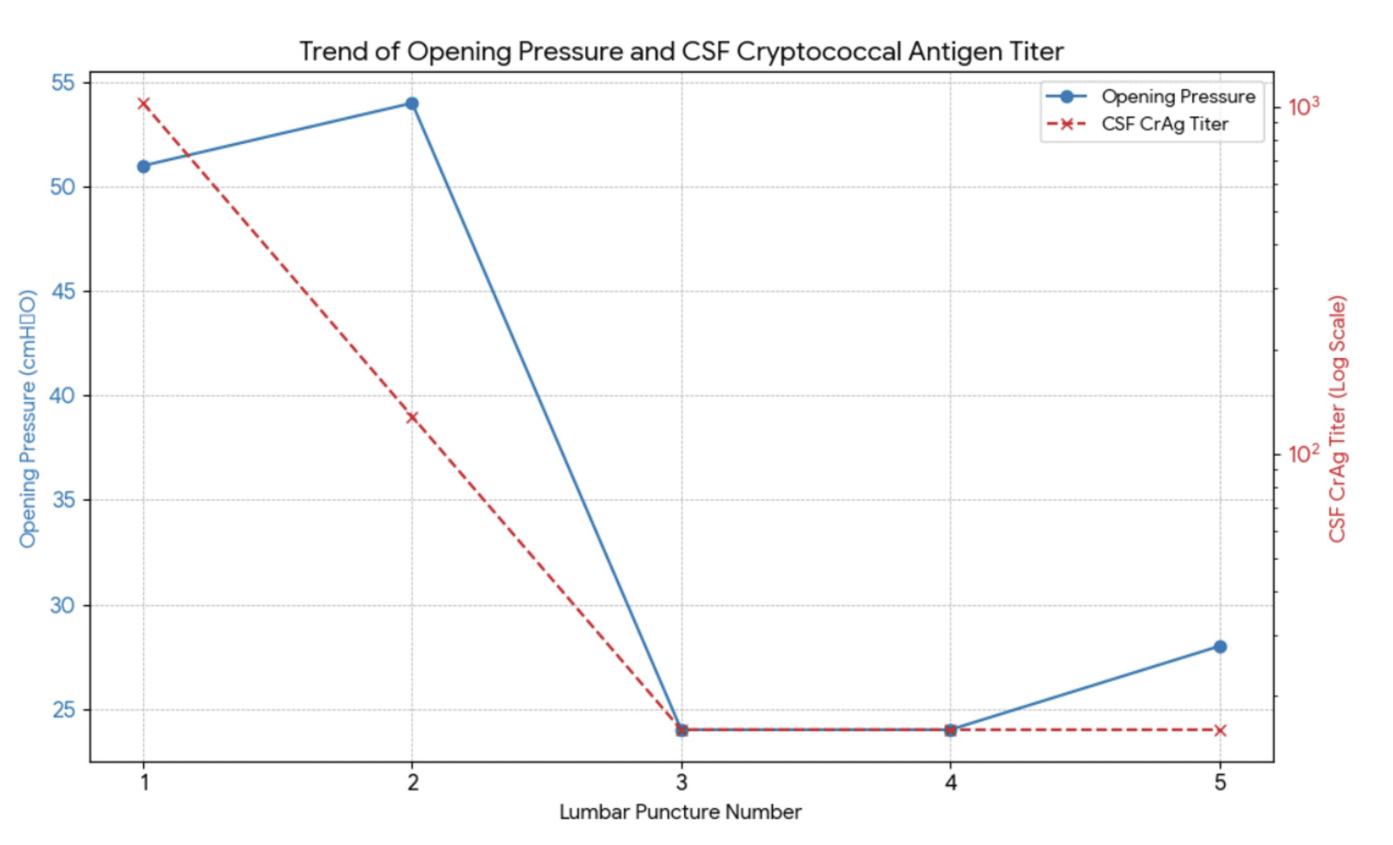
Trend of opening pressure and CSF cryptococcal antigen titer. CSF: cerebrospinal fluid; CrAg: cryptococcal antigen.

The patient was then initiated on liposomal amphotericin B at 4 mg/kg and continued with fluconazole at 1200 mg a day. This therapeutic combination was continued for two weeks, and a repeat CSF study was done. Throughout the course of the two-week therapy, the patient denied any symptoms of headache, nor was she noted to have any changes in sensorium. On the second lumbar tap, the opening pressure was 54 cmH₂O, with a closing pressure of 46 cmH₂O. A repeat quantitative CALAS (Cryptococcal Antigen Latex Agglutination System) showed a 1:128 dilution, a decrease compared to the initial 1:1024. Medical decompression was maintained using mannitol. Then, after another two weeks, at 34 days of liposomal amphotericin B, the patient had a repeat lumbar tap to check for quantitative CALAS. Opening pressure on the 3rd lumbar tap was now down to 24 cmH₂O, and repeat quantitative CALAS was now down to 1:16 dilution. Fluconazole was discontinued during this time, as the patient was documented as having prolonged QTc in the electrocardiogram. The patient completed the 28 days of high-dose fluconazole prior to discontinuation. On the sixth week of antifungal therapy, the patient had another lumbar tap for CALAS determination, which again showed a 1:16 dilution. Opening pressure was still at 24 cmH2O, the same as previously, but still asymptomatic. Figure 2 summarizes the opening pressure and CALAS trends of the patient throughout the course of therapy.

**Figure 3:**
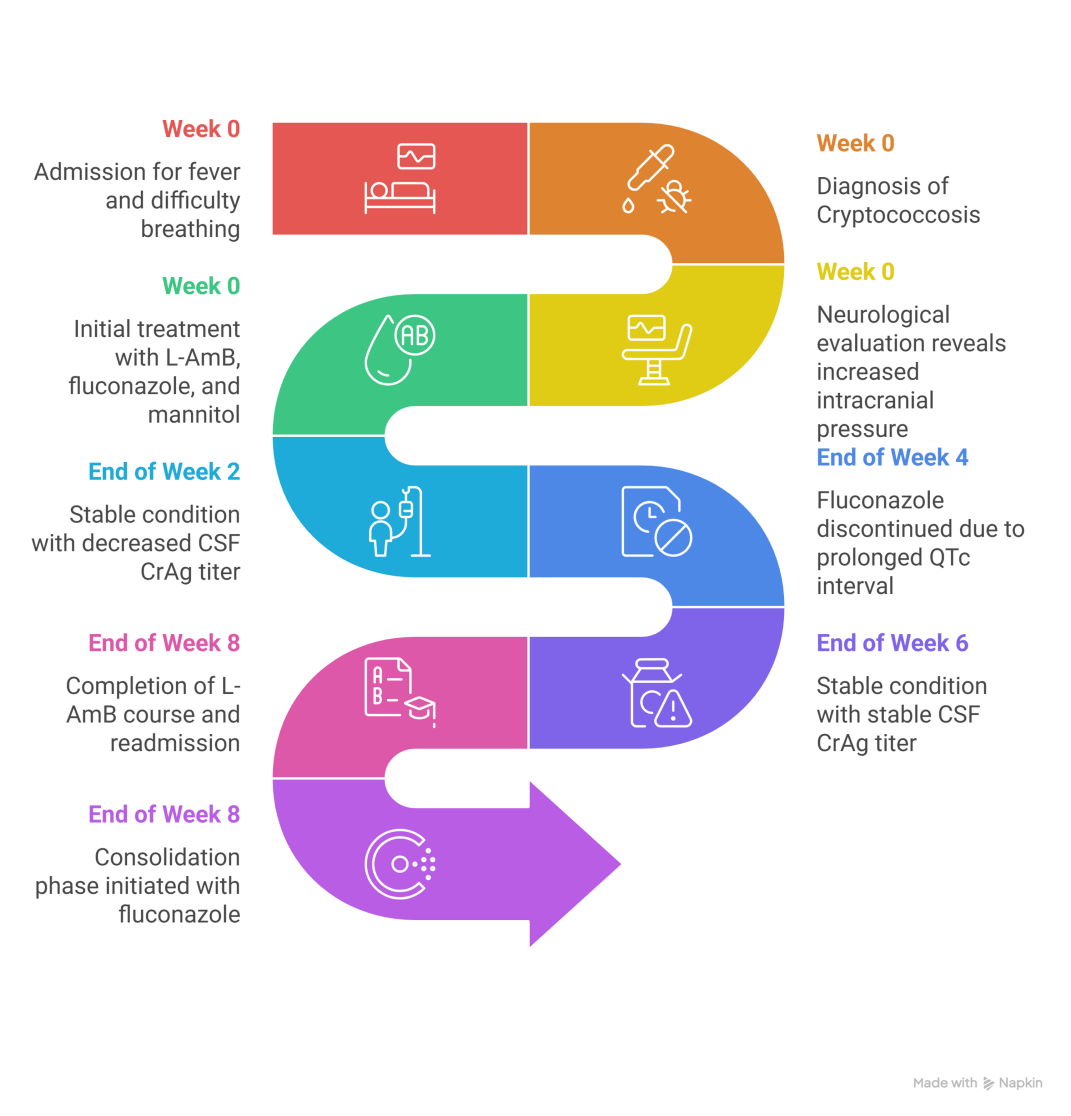
Summary of the patient's timeline of events and therapy progression. L-AmB: liposomal amphotericin B; CSF CrAg: cerebrospinal fluid cryptococcal antigen; QTc: corrected QT interval.

The patient, being clinically stable, was discharged with outpatient parenteral antimicrobial therapy (OPAT) and readmitted after completion of the eight-week course of liposomal amphotericin B, and a repeat lumbar tap was done on readmission. The opening pressure was noted at 28 cmH2O, with repeat quantitative CALAS at 1:16 dilution, the same as the previous results. The patient was then finally reinitiated with fluconazole for the consolidation phase of disseminated cryptococcal infection. The patient was clinically stable throughout the course of the therapy, with no new neurological concerns. It was also noted that HIV screening, using the point-of-care test, was conducted prior to her discharge and revealed negative results. A summary of the timeline of events since admission is shown in Figure 3.

## Discussion

CNS fungal infections may occur without a clear predisposing factor; however, they generally complicate other disease processes that impair immune function, including HIV, cancer chemotherapy, organ transplantation, severe burns, leukemia, lymphoma, other malignancies, diabetes, rheumatologic disorders, or extended corticosteroid treatment. They are designated as opportunistic. Cryptococcosis is a life-threatening opportunistic fungal infection in both HIV-positive and HIV-negative patients and is a major worldwide disseminated invasive fungal infection. Cryptococcosis, particularly in its most lethal manifestation of cryptococcal meningitis, accounts for substantial mortality and morbidity [6,7]. Cryptococcal meningitis presents with a nonspecific clinical presentation. Most instances progress subacutely, similar to other fungal diseases and tuberculosis. Typically, headaches, fever, and stiff neck are absent, and the patient exhibits symptoms of progressively escalating intracranial pressure (ICP) due to hydrocephalus (papilledema is observed in 50% of these patients) or presents with a confusional state, dementia, cerebellar ataxia, or spastic paraparesis, generally without additional focal neurological deficits. This case presents an unusual manifestation of cryptococcal meningitis in an immunocompromised individual who was taking high-dose glucocorticoid therapy and lacked the characteristic symptom of headache, which emphasizes the diagnostic dilemma linked to this condition. Although headache, fever, and altered mental status constitute the conventional triad of CNS involvement, this case shows that even their absence does not rule out CNS involvement [8].

A notable finding in this case was the consistently high ICP, despite the patient exhibiting no neurological signs or symptoms. The findings of cotton wool spots and blurred optic disc margins during the fundoscopic examination indicated possible elevated ICP, which lumbar puncture subsequently verified. Elevation of ICP in a rigid cranial cavity will trigger a number of closely interrelated events, such as reduction in the volume of the cerebral ventricles, subarachnoid space, and extracerebral CSF cisterns, distortion of the adjacent brain, and compression of the major intracranial venous sinuses [9]. These events all eventually led to secondary vascular complications such as hemorrhage and ischemia. Due to this compression, there will be alteration of the electrical and chemical properties of neurons by distortion of their architectural arrangement [10]. In this regard, corticosteroids work by reducing capillary permeability and reducing spontaneous discharge in injured nerves [11]. Corticosteroids inhibit synthesis, which in turn decreases inflammation and reduces permeability in the vasculature, causing edema. Receptors related to steroids are found in the peripheral and central nervous systems and are causative for development, differentiation of growth, and neuronal plasticity [12]. While mannitol 20% was used for this case, it is worth noting that it is not part of the standard management, and its basis was more of clinical experience and should be interpreted with discretion by the reader. However, certain studies have reported that mannitol use can alleviate symptoms of increased ICP, with one case report being used in adjunct with ventriculoperitoneal shunting [13,14].

Another important aspect in this case was the use of serum cryptococcal antigen tests, which increased our suspicion for CNS involvement. The elevated CALAS titer at 1:4906 necessitated additional examination via lumbar puncture, even if there were no neurologic symptoms. Prior research has shown that elevated serum cryptococcal antigen titers are significantly associated with CNS involvement and adverse outcomes if untreated. This case substantiates the advice that all patients with elevated blood cryptococcal antigen titers, regardless of symptomatology, should receive cerebrospinal fluid examination to assess for subclinical meningoencephalitis.

As for the management of her infection, the patient was given liposomal amphotericin B and high-dose fluconazole over an eight-week period as per international consensus guidelines. Serial lumbar punctures during the therapeutic period showed a progressive reduction in both quantitative CALAS titers and ICP, indicating therapeutic effectiveness. It is noteworthy, however, that the patient's ICP remained elevated for an extended duration during the treatment course, and this supports the significance of serial lumbar punctures in assessing treatment responses and directing therapeutic treatments.

## Conclusions

This case demonstrates the essential need to identify unusual manifestations of cryptococcal meningitis, especially in immunocompromised patients. High-dose glucocorticoid use may mask the pain caused by increased ICP by targeting multiple mechanisms of action in the CNS. Therefore, clinicians should not exclude CNS involvement in cases with markedly high serum cryptococcal antigen titers, even if there is an absence of headache. Serial lumbar punctures, comprehensive ophthalmologic evaluations, and prompt commencement of antifungal treatment are critical elements of care. A high index of suspicion for individuals exhibiting abnormal symptoms, including those who are asymptomatic, can facilitate early identification and improve patient outcomes, thereby reducing the risk of severe consequences associated with delayed treatment. Future research should investigate whether early lumbar puncture management in asymptomatic patients with elevated cryptococcal antigen titers can enhance clinical outcomes and mitigate problems associated with persistent ICP.
